# PD09 Hospital-Based Health Technology Assessment: Evaluating Skirted Aortic Valves For Transcatheter Aortic Valve Implantation

**DOI:** 10.1017/S0266462325101475

**Published:** 2025-12-29

**Authors:** Olena Filiniuk, Iryna Kostiuk, Taras Lyaskovskyi, Viktoriia Nikulina, Volodymyr Hryshchenko, Ihor Ditkivskyi, Denys Voloshyn, Volodymyr Yakovets, Mykhaylo Babenko, Kostiantyn Kosyachenko, Rabia Sucu, Laura Sampietro-Colom

## Abstract

**Introduction:**

Aortic valves for transcatheter aortic valve implantation (TAVI) vary in design. Traditional models without a skirt, used at the Amosov National Institute of Cardiovascular Surgery, have notable disadvantages. This study conducted a hospital-based health technology assessment (HB-HTA) to evaluate the feasibility of implementing skirted aortic valves to optimize resource utilization within the facility.

**Methods:**

The Amosov National Institute of Cardiovascular Surgery of the National Academy of Medical Sciences of Ukraine (the Institute) conducted this project as part of a national pilot to introduce health technology assessment (HTA) in hospitals. The institute formed a multidisciplinary team of specialists with clearly defined roles and responsibilities. The team relied on Ukrainian methodological guidelines for implementing HTA in hospitals that were approved in 2023 and were derived from recommendations from Adopting Hospital-Based Health Technology Assessment (AdHopHTA) and the Danish Centre for Evaluation and Health Technology Assessment.

**Results:**

The analysis of clinical effectiveness and safety showed that aortic valves with a skirt have advantages over those without a skirt for aortic valve replacement in patients with aortic stenosis. A systematic review of literature from PubMed and the Cochrane Library found reduced rates of paravalvular leakage (10 to 20%) and complications with skirted valves, minimizing the need for corrective interventions and further monitoring. Budget impact analysis revealed that using skirted valves reduced cost overruns and optimized resource use. Transitioning to this model required no structural changes and offered significant strategic and economic benefits.

**Conclusions:**

Considering the clinical efficacy, safety, and economic feasibility of aortic valves with a skirt, their introduction into the Institute’s practice is highly justified. These valves offer the potential to enhance long-term treatment outcomes, reduce the incidence of complications such as paravalvular leakage, and optimize resource utilization. This implementation represents a strategic step toward improving patient care and healthcare efficiency.

